# Brain Tumor Epidemiology – A Hub within Multidisciplinary Neuro-oncology. Report on the 15th Brain Tumor Epidemiology Consortium (BTEC) Annual Meeting, Vienna, 2014 

**DOI:** 10.5414/NP300846

**Published:** 2014-12-19

**Authors:** Adelheid Woehrer, Ching C. Lau, Daniela Prayer, Luc Bauchet, Myrna Rosenfeld, David Capper, Paul G. Fisher, Marcel Kool, Martin Müller, Johan M. Kros, Carol Kruchko, Joseph Wiemels, Margaret Wrensch, Heather E. Danysh, Sonia Zouaoui, Julia E. Heck, Kimberly J. Johnson, Xiaoyang Qi, Brian P. O’Neill, Samina Afzal, Michael E. Scheurer, Matthew N. Bainbridge, Darryl Nousome, Mustapha El Bahassi, Johannes A. Hainfellner, Jill S. Barnholtz-Sloan

**Affiliations:** 1 Institute of Neurology, Medical University of Vienna, Austria,; 2 Department of Pediatrics, Section of Hematology-Oncology, Baylor College of Medicine, Houston, TX, USA,; 3 Department of Biomedical Imaging and Image-guided Therapy, Medical University of Vienna, Austria,; 4 Department of Neurosurgery, CHU Montpellier, France,; 5 Service of Neurology, Hospital Clínic, University of Barcelona, Spain,; 6 German Cancer Research Center DKFZ, Heidelberg, Germany,; 7 Division of Child Neurology, Department of Neurology, Stanford University, CA, USA,; 8 SwissCore, Brussels, Belgium,; 9 Erasmus MC, Rotterdam, The Netherlands,; 10 Central Brain Tumor Registry of the US, Hinsdale, IL, USA,; 11 Department of Epidemiology and Biostatistics,; 12 Department of Neurological Surgery, University of California, San Francisco,; 13 Department of Epidemiology, University of California, Los Angeles, CA,; 14 Brown School Master of Public Health Program, Washington University in St. Louis, MO,; 15 Division of Hematology-Oncology, The Vontz Center for Molecular Studies, University of Cincinnati, OH,; 16 Department of Neurology, The Mayo Clinic, Rochester, MN, USA,; 17 Division of Pediatric Hematology/Oncology, Dalhouse University, Canada,; 18 Human Genome Sequencing Center, Baylor College of Medicine, Houston, TX, USA,; 19 Preventive Medicine, University of Southern California, Los Angeles, CA,; 20 Brain Tumor Center, University of Cincinnati, and; 21 Case Comprehensive Cancer Center, Case Western Reserve University School of Medicine, Cleveland, OH, USA

**Keywords:** brain tumor, epidemiology, clinical research, tissue-based research, risk factor research

## Abstract

The Brain Tumor Epidemiology Consortium (BTEC) is an open scientific forum, which fosters the development of multi-center, international and inter-disciplinary collaborations. BTEC aims to develop a better understanding of the etiology, outcomes, and prevention of brain tumors (http://epi.grants.cancer.gov/btec/). The 15^th^ annual Brain Tumor Epidemiology Consortium Meeting, hosted by the Austrian Societies of Neuropathology and Neuro-oncology, was held on September 9 – 11, 2014 in Vienna, Austria. The meeting focused on the central role of brain tumor epidemiology within multidisciplinary neuro-oncology. Knowledge of disease incidence, outcomes, as well as risk factors is fundamental to all fields involved in research and treatment of patients with brain tumors; thus, epidemiology constitutes an important link between disciplines, indeed the very hub. This was reflected by the scientific program, which included various sessions linking brain tumor epidemiology with clinical neuro-oncology, tissue-based research, and cancer registration. Renowned experts from Europe and the United States contributed their personal perspectives stimulating further group discussions. Several concrete action plans evolved for the group to move forward until next year’s meeting, which will be held at the Mayo Clinic at Rochester, MN, USA.

## Introduction 

The Brain Tumor Epidemiology Consortium (BTEC) is an open scientific forum that fosters the development of multi-center, international and inter-disciplinary collaborations, which shall lead to a better understanding of the etiology, outcomes, and prevention of brain tumors (http://epi.grants.cancer.gov/btec/). The Consortium was formed in 2003 after an initial meeting sponsored by the National Cancer Institute’s Division of Cancer Epidemiology and Genetics, and the National Institute of Health’s Office of Rare Diseases, and has since evolved into a self-directed consortium. BTEC holds annual meetings where working groups meet both individually and jointly to discuss current and future collaborations and scientific findings. The 2014 meeting was dedicated to the central role of epidemiology **“Brain Tumor Epidemiology – A Hub within Multidisciplinary Neuro-oncology”**; and was held in Vienna, Austria, hosted by the Austrian Societies of Neuropathology and Neuro-oncology. The program committee consisted of the local organizers Adelheid Woehrer, MD PhD and Johannes A. Hainfellner, MD, both from Vienna, Austria, in addition to the BTEC US President, Jill Barnholtz-Sloan, PhD, Cleveland, OH, Secretary Paul Fisher, MD, and Treasurer Sonia Partap, MD, both from Palo Alto, CA, USA. Meeting arrangements were made by the BTEC Meeting Manager, Ms. Bénédicte Clement, Montpellier, France. 

## Thematic workshops 

The meeting was organized in several thematic workshops with talks from a variety of renowned speakers followed by active group discussions. The opening session was held as a joint session between BTEC and the European Association of Neuro-oncology (EANO). Both organizations are dedicated to brain tumor research and may benefit from future collaboration. Two EANO representatives, **Christine Marosi, MD**, and **Wolfgang Grisold, MD**, both experienced neuro-oncologists and researchers from Vienna, Austria, co-chaired the session entitled “**Linking brain tumor epidemiology with clinical neuro-oncology”**. The first speaker, **Daniela Prayer, MD**, from Vienna, Austria spoke about modern multimodal MR-based imaging methods and emphasized the importance of metabolic and perfusion imaging. Both techniques contribute substantially not only to the delineation of primary brain tumors from other pathologies, but provide further information on intratumoral heterogeneity [1]. This information is crucial to neurosurgeons in order to target the most adequate i.e. active tumor area for biopsy [2]. Moreover, MR-based neuroradiological response patterns including pseudo-progression (post-treatment radiation effect) and pseudo-regression (disappearance of contrast enhancement following anti-angiogenic treatment) are increasingly recognized and being defined [3]. The relevance of advanced neuroimaging was further stressed by **Luc Bauchet, MD PhD**, neurosurgeon from Montpellier, France, and current BTEC Non-US President. By putting the audience in the role of a neurosurgeon, he illustrated the manifold patient-related factors and prognosticators, which need to be considered during the preoperative surgical assessment. Given his longstanding work with the French Brain Tumor Databank, he emphasized the importance of outcomes research for estimating the prognosis of an average patient. Still, he pointed towards the remaining gray areas such as the “elderly patient with glioblastoma”, for whom there is still no established treatment standard and undertreatment is a major concern [4]. He concluded that specific geriatric assessments and new endpoints such as quality-of-life or treatment toxicity measures need to be included in routine patient management. Patients with glioblastoma were also the focus of the following inspirational talk by **Myrna R. Rosenfeld, MD PhD**, neuro-oncologist from Philadelphia, PA, USA, currently based in Barcelona, Spain. Dr. Rosenfeld exemplified that the current treatment standard was established in 2005 [5] and efforts to improve upon it have not yet yielded a new standard of care. Nevertheless, studies suggest that patients with glioblastoma are living longer [6], which is most likely due to improved management of co-morbidities and an understanding that improved quality of life often translates into improved overall survival. Thus, clinical trials should incorporate guidelines for uniform management of co-morbidities and a battery of patient-reported outcomes. These data are important because the determination of the success of a trial should not be based only on statistical significance but clinical significance. She further emphasized that we need to understand the barriers to dissemination and acceptance of new treatment regimens into routine practice, including those validated by robust phase III studies. 

After this clinically oriented session, the group turned towards molecular genetic analyses of primary brain tumors. The following session entitled “**Linking brain tumor epidemiology with tissue-based research”** was co-chaired by **Matthias Preusser, MD**, from Vienna, Austria, and **Margaret Wrensch, MPH PhD**, from San Francisco, CA, USA. The session included three talks followed by the keynote. **Jill Barnholtz-Sloan, PhD**, from Cleveland, OH, USA, shared novel insights into The Cancer Genome Atlas (TCGA) project, which set out to fully describe the molecular characteristics of over twenty different types of cancer, including lower grade gliomas (WHO grade II and III) and glioblastoma (WHO grade IV). Two comprehensive papers on glioblastomas have been published from TCGA GBM working group, which showed that distinct gene expression- and DNA methylation-based subtypes exist, but are not necessarily associated with clinical outcomes [7, 8]. GBM patients who have a combination of the proneural gene expression subclass, hypermethylator phenotype (CpG island methylator phenotype or G-CIMP) and *IDH1* mutation appear to have improved survival (~ 8 – 10% of all patients), offering some guidance for this subpopulation. In line, preliminary data from TCGA LGG working group suggests that 3 distinct molecular subtypes of LGG exist regardless of histology (astrocytoma, oligodendroglioma or oligo-astrocytoma) or WHO grade (II or III). These subtypes are defined by *IDH1* mutation status combined with 1p/19q deletion status. Updated information on publications from TCGA working groups can be found here: http://cancergenome.nih.gov/cancersselected. Dr. Barnholtz-Sloan’s talk was complemented by a subsequent lecture by **David Capper, MD**, a young neuropathologist from Heidelberg, Germany. He has a particular expertise in the molecular classification of brain tumors and is especially interested in the translation of novel markers into routine diagnostic use. Of note, Dr. Capper introduced an ambitious project of his research group concerning the construction of a comprehensive reference library for primary brain tumors based on DNA methylation profiles of prototypic examples. The project has already advanced to a pre-final stage and they hope to be able to launch it within the next months. By then the reference library shall be open via a web-based tool to all neuropathology/neuro-oncology centers worldwide allowing for direct comparison of the methylation profiles of diagnostically challenging cases with those of the reference set. He pointed out that, by complementing traditional histopathology with genome-wide DNA methylation profiles, diagnostic accuracy can likely be increased. However, at the same time, the diagnostic significance of histopathology as the long-established gold standard is challenged. This touched on a sensitive point and stimulated an active discussion with supporting and opposing opinions. However, the key role of neuropathologists as caretakers of the tumor tissue evolved as a common denominator. After the first two talks, the focus turned towards pediatric brain tumors. **Paul Fisher, MD PhD**, from Palo Alto, CA, USA, provided an overall introduction to pediatric brain tumors and then transitioned to the keynote talk of this session **“Transition from genotypes to epigenotypes of brain tumors”** which was given by **Marcel Kool, PhD**, cancer biologist at DKFZ Heidelberg, Germany. Both provided a comprehensive overview of the most relevant molecular markers in pediatric brain tumors and their impact on pediatric neuro-oncology. Also, pediatric brain tumors might display molecular heterogeneity as best evidenced by medulloblastoma, one of the most common malignant brain tumors in children. Dr. Kool and co-workers contributed considerably to the identification of distinct molecular subgroups, i.e., WNT, SHH, Group 3, and Group 4 tumors, which differ not only in terms of gene expression profiles and pathogenic pathways but also in terms of patient prognosis [9, 10]. Molecular subtyping is important and increasingly recognized for patient stratification into low-, average-, and high-risk groups in order to minimize treatment effects and maximize cure rates. One of Dr. Kools’s declared aims is to find optimal therapeutic targets for the respective subgroups – an ambitious aim, which seems almost within reach for WNT and SHH-driven tumors, as small-molecule pathway inhibitor drugs are on their way into clinics [11]. Beyond medulloblastoma, Dr. Kool and his group are currently working on the entire group of embryonal brain tumors. In fact, DNA methylation and copy number profiling have changed our concept of CNS primitive neuroectodermal tumors. In particular, scientific evidence suggests that embryonal tumor with abundant neuropil and true rosettes, ependymoblastoma, and medulloepithelioma share molecular similarity and comprise a single clinicopathological entity [12]. Numerous questions arose and prompted discussions on new therapeutic avenues in neuro-oncology; towards “personalized medicine” of patients with brain tumors. 

As fundraising and sustainability is an important issue for BTEC, the subsequent workshop focused on the **European funding perspective**. **Martin Müller**, European Advisor for Research at SwissCore was invited to provide an overview on funding opportunities within the European Framework Programme for Research and Innovation, Horizon 2020 (http://ec.europa.eu/programmes/horizon2020/). As such it is the world’s largest program supporting scientific research with a total budget of around € 80 billion for 2014 – 2020, and covers all areas of science from fundamental research to commercialization of new products and services. In the field of brain tumor epidemiology, the European Research Council offers prestigious grants to excellent researchers in different phases of their careers. The grants are for up to € 3.5 million for 5 years for a single Principal Investigator. These grants are, however, very competitive, with an average success rate of 12%. Activities in the sub-program area “health, demographic change, and wellbeing” focus on interdisciplinary collaborative research projects aimed at delivering solutions to concrete problems in the field of health that would have societal and economical impact. Finally, a last instrument worth noting is the European Cooperation in Science and Technology (COST) that offers funding for cooperative activities such as the organization of conferences, summer schools and short visits. 

**Carol Kruchko**, President and Founder of the Central Brain Tumor Registry of the United States (CBTRUS), Chicago, IL, chaired the last workshop entitled **Linking brain tumor epidemiology with cancer registration**. The session was opened by **Johannes A. Hainfellner, MD**, Vienna, Austria, who summarized the key-steps in the translation of biomarkers from basic research to routine diagnostic use [13, 14]. Special emphasis was put on analytical and clinical test performances of the various biomarkers including *IDH1* mutation, *MGMT* promoter methylation, and 1p/19q chromosomal status. Most importantly, the WHO brain tumor histopathological diagnosis is a crucial prognosticator, as each brain tumor type is associated with a distinct biological behavior and patient prognosis. Dr. Hainfellner further introduced the changing concept of modern neuropathology as the caretaker of tumor tissue and party responsible for the assignment and conducting of appropriate molecular tests. This concept was further refined by **Johan M. Kros, MD PhD**, from Rotterdam, The Netherlands, who shared the enormous expertise he gained during his longstanding work as reference neuropathologist for the EORTC brain tumor group. Over the years Dr. Kros was involved in many EORTC trials and acquired firsthand experience about the importance of central histopathology reviews and the magnitude of inter-observer variability [15]. Dr. Kros called for the development of a concise, evidence-based diagnostic algorithm for diffuse gliomas [16]. **Hiroko Ohgaki, PhD**, Head, Section of Molecular Pathology at the International Association for Research on Cancer (IARC), Lyon, France, reported on the IARC’s perspective on cancer registration. One of their major duties is to edit and publish the “WHO Classification of Tumors” series including the “WHO Classification of Tumours of the Central Nervous System”. Long anticipated, she informed the group about the status of the planned update for the current fourth edition of WHO Classification of Tumors of the Central Nervous System and disclosed the key dates during the preparation phase. 

## Abstract session 

A total of 13 abstracts were submitted from US and European research groups and reflected the broad spectrum of interests, which ranged from risk factor research to genetic and descriptive epidemiology. Two abstracts were awarded with Young Investigator Awards supported by the American Brain Tumor Association (www.abta.org); **Heather E. Danysh, MHS**, Houston, TX, USA, for her paper on “A statewide assessment of childhood central nervous system tumors and traffic-related air pollution” and **Sonia Zouaoui**, **PhD**, Montpellier, France, who presented data on the “Descriptive epidemiology of 13,038 newly diagnosed and histologically confirmed meningiomas in France: 2006 – 2010“. Dr. Danysh focused on a topic of growing concern i.e., exposure from traffic-related air pollution and childhood brain tumor risk. Based on the results of a large population-based assessment in Texas she found positive associations between area-level 1,3-butadiene and DPM levels and astrocytoma incidence as well as between DPM levels and medulloblastoma incidence. Dr. Danysh concluded that future assessments would need to consider additional pollutants as well as exposure misclassification. In contrast, Dr. Zouaoui presented the latest research data of the French Brain Tumor Databank. With more than 13,000 meningioma cases overall, this study constitutes the most comprehensive descriptive epidemiological study on meningiomas, so far, allowing for detailed subgroup analysis for the individual morphological subtypes [17]. She found a slight but significant increase in the incidence rate of atypical meningiomas after 2007. Further abstract highlights included the work of **Julia E. Heck, PhD**, Los Angeles, CA, USA, who assessed differences of brain tumor incidence between Hispanic and White children. Based on data linkage between the birth registry and the California Cancer Registry, her preliminary data suggest that for most CNS tumor types including astrocytoma, ependymoma, and medulloblastoma, first generation US Hispanic children experience the lower tumor rates of their parent countries, while second generation children begin to lose the advantage of immigration, and their rates begin to rise approximating those of white children. **Kimberly Johnson**, **MPH PhD, and BTEC Secretary-Elect** from St. Louis, MO, USA evaluated exome sequence data from 267 glioblastoma and 223 low-grade glioma cases from TCGA for germline loss-of-function variants in genes associated with familial cancer syndromes. Her results support prior evidence for the involvement of the *MSH6* gene in the predisposition to brain tumors. She concluded, however, that germline loss-of-function variants in known familial cancer syndrome genes might be only rarely involved in glioma susceptibility in the general population of glioma cases. Dr. Johnson further concluded that allelic imbalance analyses could inform on the functional relevance of rare variants in tumor suppressor genes. **Qi Xiaoyang, PhD**, Cincinnati, OH, USA, presented follow-up data on SapC-DOPS nanovesicles, assembled from the naturally occurring protein saposin C (SapC) and the membrane lipid dioleylphosphatidylserine (DOPS), which were shown to selectively target and effectively kill diverse cancer cells in vitro and in vivo as a consequence of their binding affinity to phosphatidylserine [18]. They were now able to show that phosphatidylserine is overexpressed on surface membranes of brain tumor cells and tumor-associated vasculature, thereby rendering it a druggable target. **Ching Lau, MD PhD and BTEC US Vice President-elect,** Houston, TX, USA presented on behalf of an international consortium on recent findings on intracranial germ cell tumors (IGCTs), which are rare and biologically diversified tumors affecting mainly male adolescents with the highest incidence in Japan and other Asian countries. Previously they reported that the KIT/RAS and AKT/mTOR signaling pathways are frequently mutated in IGCTs [19]. In addition to somatic mutations, they observed significant enrichment of novel and rare germline variants in the *JMJD1C* gene, which were strongly associated with the risk of developing IGCT (odds ratio of 4.8). *JMJD1C* is a histone demethylase and coactivator of the androgen receptor shown to be required in the development and maintenance of germ cells in mice and helps maintain pluripotency in ESCs by sustaining the expression of miR302. **Adelheid Woehrer, MD PhD**, BTEC Non-US Vice President, Vienna, Austria, reported on behalf of the Austrian Brain Tumor Registry on the incidence and survival of patients with primary CNS lymphoma in Austria. Based on median overall survival rates of only 11.0 months they found that the prognosis has remained unchangedly poor at the population level across different countries and decades; however, a fraction of ~ 20% of the patients, who are still alive at 5 years after diagnosis, gives reason for cautious optimism as it renders the disease “potentially curable”. Primary CNS lymphoma was also the topic of **Brian Patrick O’Neill’s** talk, **MD,** from Rochester, MN, USA, who described recurrent genetic alterations, which correlate with survival and might yield clues to pathogenesis. Several abstracts focused on malignant gliomas including epidemiological findings on diffuse intrinsic pontine gliomas in children by **Samina Afzal, MD**, from Halifax, Canada, the role of chicken pox and shingles in glioma risk (**Michael E. Scheurer, PhD MPH,** TX, USA), the relevance of shelterin complex genes in glioma susceptibility (**Matthew N. Bainbridge, PhD,** Houston, TX, USA), the expression quantitative trait loci and allele-specific expression of genome-wide association study variants in glioma (**Darryl Nousome, PhD**, Los Angeles, CA, US), as well as the importance of circulating mutant DNA to assess glioblastoma dynamics by **El Mustapha Bahassi, PhD,** Cincinnati, OH, USA. 

## Conclusions 

**Figure 1:**
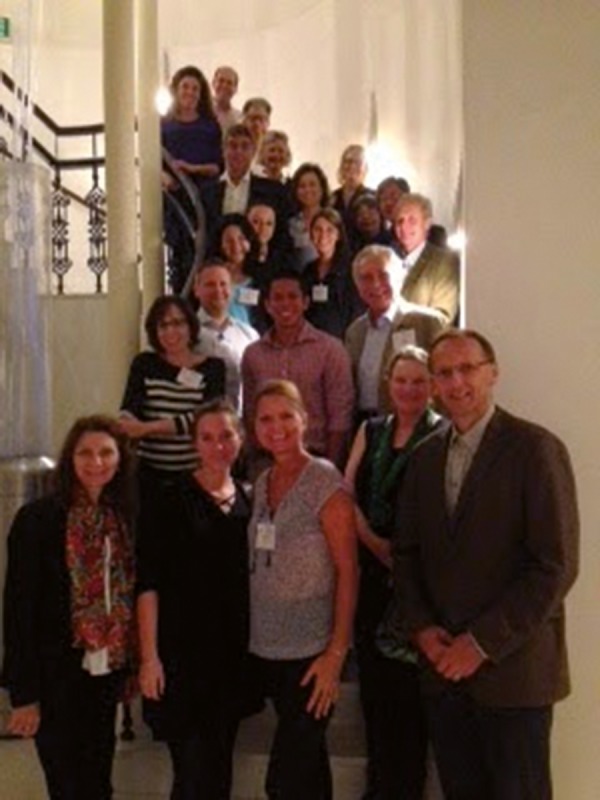
BTEC group photo 2014.

The BTEC meeting participants covered all major aspects of brain tumor epidemiology in a two-day meeting in Vienna, Austria. Even after intense working days, the city of Vienna had much to offer. The group spent a wonderful dinner at a traditional Viennese Heuriger in the vineyards close-by. And Johannes was a generous host and organized an unforgettable post-congress tour with his own personal note for those with a special interest in Viennese medical history and music (Figure 1). Intense but invaluable 2 days! 

## Acknowledgments 

This meeting would have not been possible without the generous support of our sponsors from Europe: Austrian Society of Neuropathology, Austrian Society of Neuro-Oncology, Comprehensive Cancer Center of the Medical University of Vienna, Roche Austria, Dako – An Agilent Technologies Company; and the United States: National Cancer Institute at the National Institutes of Health, National Brain Tumor Society, Pediatric Brain Tumor Foundation and American Brain Tumor Association. 

## Conflict of interest 

All authors declare that there are no conflicts of interest.
